# Membrane Binding, Cellular Cholesterol Content and Resealing Capacity Contribute to Epithelial Cell Damage Induced by Suilysin of *Streptococcus suis*

**DOI:** 10.3390/pathogens9010033

**Published:** 2019-12-30

**Authors:** Désirée Vötsch, Maren Willenborg, Walter M.R. Oelemann, Graham Brogden, Peter Valentin-Weigand

**Affiliations:** 1 Institute for Microbiology, University of Veterinary Medicine Hannover, 30173 Hannover, Germany; desiree.voetsch@tiho-hannover.de (D.V.); maren.willenborg@tiho-hannover.de (M.W.);; 2 Departamento de Imunologia, Instituto de Microbiologia Paulo Góes, Universidade Federal do Rio de Janeiro (UFRJ), 21941-901 Rio de Janeiro, Brazil; 3 Department of Physiological Chemistry, University for Veterinary Medicine Hannover, 30559 Hannover, Germany; graham.brogden@tiho-hannover.de

**Keywords:** *Streptococcus suis*, suilysin, pore-forming toxin, membrane repair, respiratory epithelial cells, cholesterol-dependent pore-forming cytolysin

## Abstract

*Streptococcus* (*S.*) *suis* is a major cause of economic losses in the pig industry worldwide and is an emerging zoonotic pathogen. One important virulence-associated factor is suilysin (SLY), a toxin that belongs to the family of cholesterol-dependent pore-forming cytolysins (CDC). However, the precise role of SLY in host–pathogen interactions is still unclear. Here, we investigated the susceptibility of different respiratory epithelial cells to SLY, including immortalized cell lines (HEp-2 and NPTr cells), which are frequently used in in vitro studies on *S. suis* virulence mechanisms, as well as primary porcine respiratory cells, which represent the first line of barrier during *S. suis* infections. SLY-induced cell damage was determined by measuring the release of lactate dehydrogenase after infection with a virulent *S. suis* serotype 2 strain, its isogenic SLY-deficient mutant strain, or treatment with the recombinant protein. HEp-2 cells were most susceptible, whereas primary epithelial cells were hardly affected by the toxin. This prompted us to study possible explanations for these differences. We first investigated the binding capacity of SLY using flow cytometry analysis. Since binding and pore-formation of CDC is dependent on the membrane composition, we also determined the cellular cholesterol content of the different cell types using TLC and HPLC. Finally, we examined the ability of those cells to reseal SLY-induced pores using flow cytometry analysis. Our results indicated that the amount of membrane-bound SLY, the cholesterol content of the cells, as well as their resealing capacity all affect the susceptibility of the different cells regarding the effects of SLY. These findings underline the differences of in vitro pathogenicity models and may further help to dissect the biological role of SLY during *S. suis* infections.

## 1. Introduction

Suilysin (SLY), the main cytolysin of *Streptococcus* (*S.*) *suis*, is a member of the family of cholesterol-dependent cytolysins (CDC), which are mainly produced by Gram-positive bacteria [1]. The soluble monomeric protein consists of four domains (D1–D4), with each domain playing an important role in the process of pore-formation. After binding to areas enriched in cholesterol and sphingolipids (lipid rafts) on the host cell membrane, the monomers form an oligomer and further conformational changes lead to a ring- or arc-shaped prepore-complex. Then, D2 collapses and the transmembrane β-hairpins of D3 insert into the membrane, building a large transmembrane β-barrel pore. This pore, consisting of ~35–50 monomers, has a diameter of approximately 30 nm and allows the passage of macromolecules and ions [1,2,3]. Next to the formation of these so-called macropores, small and medium sized pores are formed simultaneously in a dynamic manner [4]. Pore formation contributes to the pathogenicity of *S. suis* as it leads to depolarization, impaired (immune) response, and, eventually, death of the affected host cell [5,6].

The trypthophan-rich undecapeptide in D4 harbors a cholesterol recognition motif (CRM) for recognition and binding of the toxin to cholesterol [7,8]. Nevertheless, it is still discussed controversially whether cholesterol itself functions as the only membrane receptor for SLY or whether other receptor(s) exist. For instance, some members of the CDC family, such as intermedilysin, vaginolysin, and lectinolysin, are specific for human cells due to their binding to human CD59, a GPI-anchored protein [9,10,11]. However, they still need cholesterol to anchor to the host cell membrane during pore formation [12,13,14].

SLY was identified by Jacobs et al. [15] and is a virulence-associated factor of *S. suis* [15,16,17,18,19], an emerging zoonotic pathogen which leads to high economic losses in the pig industry by causing several inflammatory diseases in swine. Interestingly, SLY is present in the majority of virulent European and Asian *S. suis* strains, but is less frequent in the North American strains [20,21,22]. SLY can also be expressed by avirulent strains [17] and, on the other hand, virulent strains lacking the *sly*-gene exist [23]. Nevertheless, SLY contributes to adherence and invasion of *S. suis* [18,24,25] and reduces complement-dependent killing and phagocytosis of the pathogen [26,27]. Furthermore, SLY induces changes in the host cell cytoskeleton [28] and the release of pro-inflammatory and immunomodulatory cytokines and chemokines, such as IL-6, IL-8, TNF-α, and IL-10 [29,30,31,32].

Studies on *S. suis* and SLY have been conducted in several cell lines and primary cells [3,18,33]. Both cell types provide different advantages and disadvantages. Cell lines are easy to culture and manipulate, they are cost-effective, have a longer life span, and a lower variability compared with primary cells. Additionally, in contrast to primary cell cultures, they are usually not contaminated with other cell types like fibroblasts or microorganisms. However, misidentified and contaminated cell lines have led to doubtful results in the past [34,35]. A disadvantage is that cell lines differ genetically and phenotypically from the in vivo target cell(s) [36,37]. In contrast, primary cells are more difficult to handle but show morphology and functions that mimic much more closely the in vivo conditions.

Besides the fact that cell lines and primary cells vary in their morphology and functions, other factors contributing to the susceptibility of cells towards cytolysins should be considered. One important factor is the binding affinity of the cytolysin for different cell types, which depends on the membrane composition, the receptor expression, as well as the host species origin. Furthermore, the calcium influx provided by the formation of a permeable pore, the toxin oligomerization, as well as membrane repair mechanisms of the cell have an impact on the sensitivity of cells to damage caused by cytolysins [38].

The objective of our study was to investigate the susceptibility of different respiratory epithelial cells lines and primary cells towards the *S. suis* cytolysin SLY and possible factors contributing to their susceptibility.

## 2. Results and Discussion

### 2.1. Time- and Dose-Dependent Damage in Different Respiratory Epithelial Cells Caused by SLY

**Figure 1:**
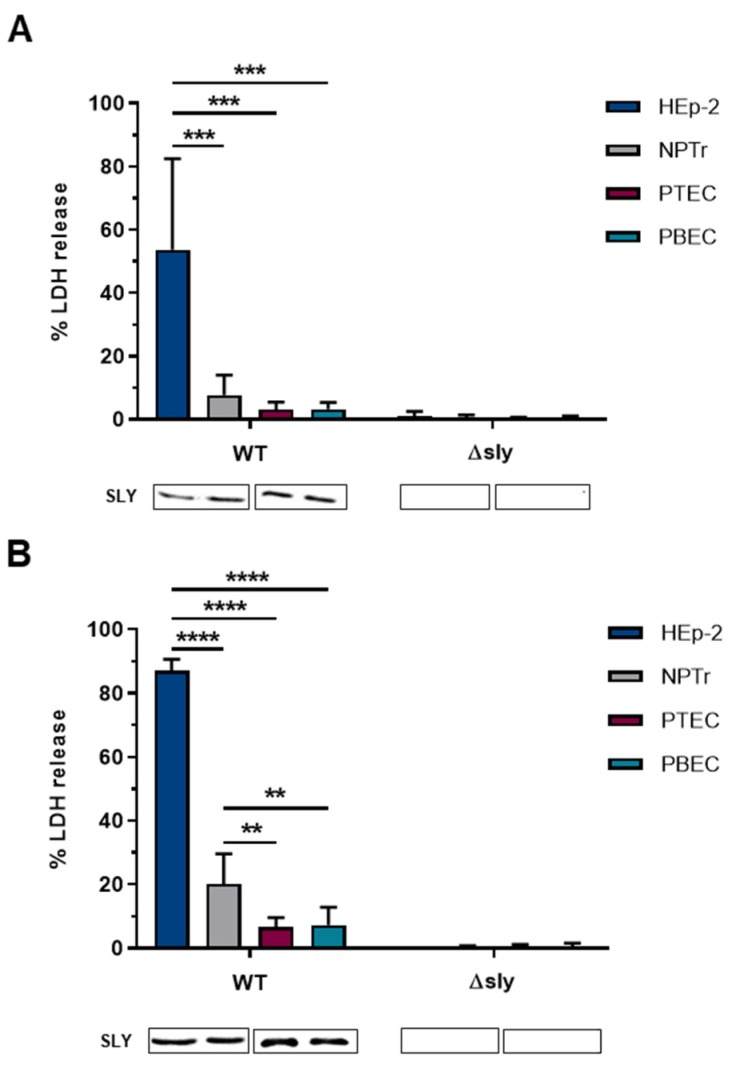
HEp-2 and NPTr cells, as well as PTEC and PBEC, were incubated with *S. suis* wild-type (WT) strain 10 and its SLY-deficient mutant (∆*sly*) at MOI 100:1 for (**A**) 2 h and (**B**) 4 h at 37 °C. After incubation, cytotoxicity was measured by LDH release assay. Results are expressed as percentage LDH release and mean ± SD of at least three independent experiments are shown. Significant differences between the cell types are indicated by ** *p* < 0.01, *** *p* < 0.001, and **** *p* < 0.0001; two-way ANOVA followed by Tukey post-hoc test. Below the graph, an immunoblot analysis for detection of SLY-expression in the supernatant of infected cells is shown.

*S. suis* is known to induce cell damage in various host cells [3]. Since respiratory epithelial cells represent a first barrier against *S. suis* infections, we first compared the susceptibility of two different immortalized epithelial cell lines derived from the respiratory tract, the human epithelial cell line (HEp-2), and the newborn pig tracheal epithelial cell line (NPTr) towards the cytotoxic effects of an infection with *S. suis*. For this, we incubated both cell lines with a virulent SLY-positive *S. suis* serotype 2 wild-type (WT) strain and its isogenic SLY-deficient mutant (∆sly) at MOI 100:1 at 37 °C for 2 h (Figure 1A) and 4 h (Figure 1B), respectively. Cell damage was determined using a lactate dehydrogenase (LDH) release assay. As expected, we found that damage of HEp-2 and NPTr cells caused by *S. suis* is dependent on SLY since no cytotoxicity was detected when cells were incubated with the mutant strain lacking the *sly*-gene. Immunoblot analysis of the supernatant of the infected cells confirmed the absence of SLY (Figure 1A,B, lower part). Moreover, SLY-induced cell damage was time-dependent since the LDH release measured after 4 h was higher than after 2 h. This can be explained by the fact that bacteria produce and secrete SLY while they are replicating during cell incubation [15,39]. Most likely, SLY-molecules accumulate on the cell surface over time, thereby inducing more and/or larger pores which are responsible for higher cytotoxic effects after 4 h of infection. Those results are in good agreement with other studies in which SLY-induced cell damage was dependent on the incubation time and/or bacterial number. Comparable results were found in HEp-2 cells [18], porcine kidney epithelial cells (LLC-PK1) [33], human (HBMEC) and porcine brain microvascular epithelial cells (PBMEC) [29,40], porcine choroid plexus epithelial cells (PCPEC) [41], human astrocytes [42], porcine neutrophils [27], a murine macrophage cell line (J774) [43], and porcine bronchial epithelial cells differentiated under air-liquid interface conditions [44,45]. Interestingly, we observed significantly greater damage in *S. suis*-infected HEp-2 cells than in infected NPTr cells and this phenotype was independent of the incubation time. The amount of SLY in the supernatant of both infected cell types was comparable (Figure 1A,B, lower part) and no differences in bacterial growth of the investigated strains could be observed during the whole experiment (Figure S1). Hence, HEp-2 cells seem to be more susceptible to the cytotoxic effects of SLY compared to NPTr cells.

Next, we investigated the effects of *S. suis* on primary respiratory epithelial cells isolated from the trachea (PTEC) and bronchi (PBEC) of pigs. Undifferentiated PTEC and PBEC were incubated at 37 °C with the strains described above at MOI 100:1 for 2 h (Figure 1A) and 4 h (Figure 1B), respectively. Notably, the cell damage in the undifferentiated primary cells was even lower than in NPTr cells and the LDH release did not significantly increase during longer incubation time, despite a bacterial number corresponding to that present in the infection of NPTr and HEp-2 cells (Figure S1). Furthermore, we did not observe any differences between cells from the upper (PTEC) and lower (PBEC) respiratory tract. This is in contrast to findings in our previous study in which we found PBEC to be more susceptible than PTEC [44]. However, in the previous study, PTEC and PBEC had been differentiated under air-liquid interface conditions, which might affect the susceptibility of the cells due to different levels of differentiation.

**Figure 2:**
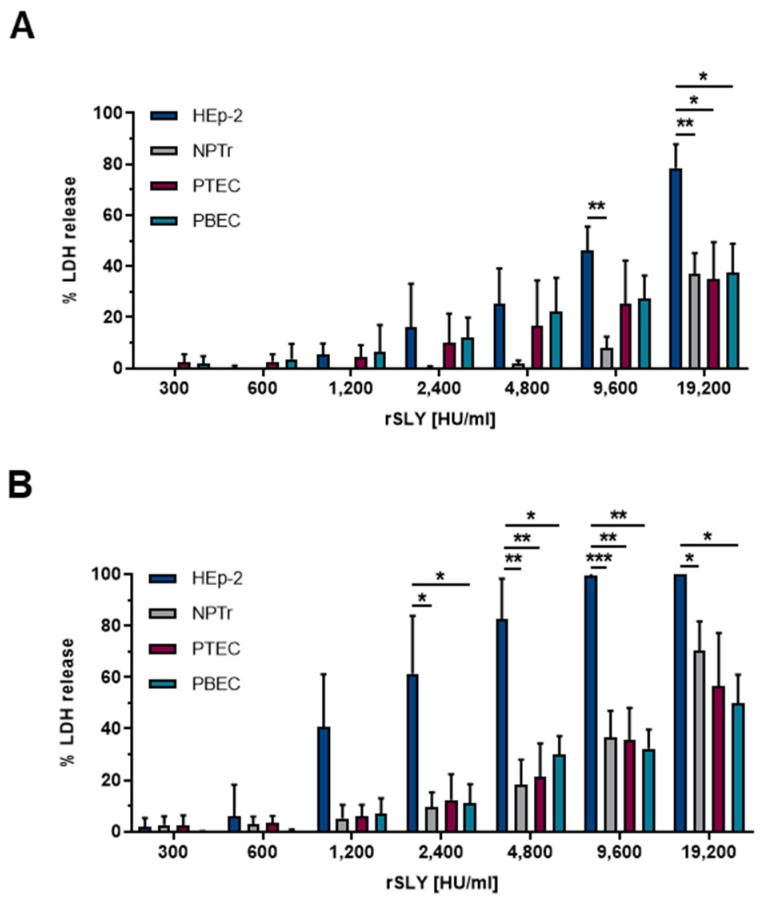
HEp-2 and NPTr cells, as well as PTEC and PBEC, were treated with 300–19,200 HU/mL recombinant SLY (rSLY) for (**A**) 2 h and (**B**) 4 h at 37 °C. Cytotoxicity was measured by LDH release assay. Results are expressed as percentage LDH release and mean ± SD of at least three independent experiments are shown. Significance is indicated by * *p* < 0.05, ** *p* < 0.01, and *** *p* < 0.001; two-way ANOVA followed by Tukey post-hoc test.

To analyze SLY-induced cell damage in more detail, we treated the cell lines as well as undifferentiated PTEC and PBEC with purified recombinant SLY (rSLY) in order to analyze effects at defined concentrations of the toxin. We decided to specify the concentration of the rSLY in HU/mL to allow a direct comparison with the cytotoxic activity of other CDC. Cells were incubated with 300 HU/mL up to 19,200 HU/mL rSLY for 2 h (Figure 2A) and 4 h (Figure 2B), respectively, at 37 °C and cell damage was determined by measuring the release of LDH. In all cell types, we found a dose-dependent cell damage induced by rSLY, which is in good agreement with other studies performed in HEp-2 cells and HBMEC [25,46]. Consistent with our findings in the infection experiment described above, HEp-2 cells showed a significantly higher level of cell damage induced by rSLY when compared to NPTr cells and the primary porcine respiratory epithelial cells. To the best of our knowledge, there are no reports on a direct comparison of the SLY-susceptibility between the human HEp-2 cells and porcine cells. However, some studies about the susceptibility of other cell types towards other CDC members have been reported. Tanigawa et al. tested the sensitivity of macrophages towards the CDC streptolysin O (SLO) of *S. pyogenes* and found immature myeloid cell lines to be sensitive towards the toxic effects, whereas mature macrophage cell lines were resistant [47]. Similar findings were reported for two different human monocyte cell lines (U937 and THP-1) [46]. In the latter study, U937 cells showed a higher sensitivity towards the CDC pneumolysin (PLY) of *S. pneumoniae* and the authors hypothesized that this might be due to the abundance of cholesterol-glycolipid rafts on the plasma membrane. Additionally, this group tested the effect of PLY on two different human pulmonary epithelial cell lines (A549 and L132), however those cells showed the same sensitivity to PLY [48]. In another study, cytotoxic effects of an infection with *S. suis* serotype 2 to human meningeal cells and human astrocytes were evaluated and they found that astrocytes were generally more sensitive towards cytotoxic effects of SLY [42]. Thus, different cell lines even originating from the same tissue type were found to be differently susceptible towards CDCs. Nevertheless, the factor(s) contributing to these differences have not been clarified.

In addition, the importance of the host cell tissue origin remains unclear. One might assume that NPTr cells and the primary cells react similarly in the presence of SLY as they are both derived from pigs. However, we found that human HEp-2 cells were more sensitive than porcine cells. Notably, studies on PLY revealed different sensitivities of human, mouse, and guinea pig cells, which might be explained by an inhibitory apolipoprotein (CH-ApoB-100) which is only present in mice [49]. Nevertheless, for HEp-2 cells, it has to be noted that these cells were mistakenly assumed to represent respiratory cells (originating from the larynx). However, according to a recent classification by the ATCC^®^, HEp-2 cells were established from a HeLa cell contamination, which are cervical epithelial cells. In contrast, NPTr cells were generated by serial culture of primary tracheal epithelial cells from a newborn pig [50]. They show a more primary cell phenotype since they possess the ability to develop a multilayer, containing ciliated and mucus-producing cells when cultured under air-liquid interface conditions [51]. Like NPTr cells, PTEC and PBEC originate from the primary host species pig and, thus, represent the main target cells of *S. suis*. Moreover, those cells are not immortalized, but are used directly after isolation from the respective tissue. Since they are able to develop an epithelial barrier consisting of a multilayer of basal cells, ciliated, and mucus-producing cells under air-liquid interface conditions [44,45], PTEC and PBEC are very closely related to respiratory cells present in vivo.

Regarding the susceptibility of the cells from the upper (PTEC) and lower porcine respiratory tract (PBEC), no significant differences were observed, independent of the toxin concentration, confirming the results of the *S. suis*-infection experiment.

Interestingly, in all tested cell types, the amount of released LDH was higher after treatment for 4 h, even though cells were treated with the same toxin concentrations as they were for 2 h. One explanation could be that the interaction of SLY with cells is a dynamic process leading to the “opening and closing” of pores due to an iterative process of pore formation and pore removal by the host cell [52,53,54,55]. Cells are able to reseal cytolysin-induced pores in the host cell membrane and until a critical toxin concentration is reached, cell lysis is efficiently prevented [52,56]. Furthermore, pores of different sizes or incomplete pores (arcs) are built [2,57,58,59,60], allowing the passage of molecules with varying sizes and triggering different cellular responses. We assume that cells can recover more efficiently or even completely from the damage induced by SLY during 2 h of incubation, but when incubated for 4 h, they may not compensate the damaging effects of SLY any more. Taken together, we found time- and dose-dependent damage induced by SLY in all tested cell types, of which the human epithelial cell line HEp-2 showed the highest susceptibility towards SLY.

### 2.2. The Amount of Membrane-Bound SLY Is Not the Sole Factor Contributing to Cellular Damage and Is Not Only Dependent on the Total Cellular Cholesterol Content

**Figure 3:**
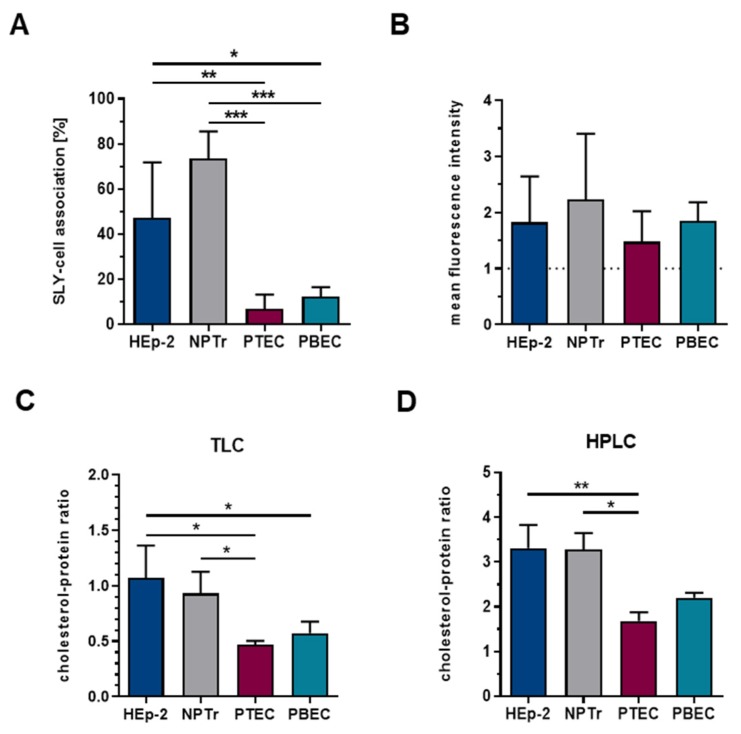
HEp-2 and NPTr cells, as well as PTEC and PBEC, were treated with 120 HU/mL rSLY for 2 h at 37 °C. Binding of SLY was analyzed using flow cytometry analysis. Results are expressed as (**A**) percentage SLY-cell association and (**B**) mean fluorescence intensity (normalized to control cells, indicated by the dashed line). Mean ± SD of at least three independent experiments are shown. Significance is indicated by * *p* < 0.05, ** *p* < 0.01, and *** *p* < 0.001; one-way ANOVA followed by Tukey post-hoc test. Cellular cholesterol content of HEp-2 and NPTr, as well as of PTEC and PBEC was analyzed using (**C**) TLC and (**D**) HPLC. The cholesterol content was related to total cellular protein content (determined by BCA). Results are expressed as the cholesterol–protein ratio. Mean ± SD of at least three independent experiments are shown. Significance is indicated by * *p* < 0.05 and ** *p* < 0.01; one-way ANOVA followed by Tukey post-hoc test.

Since the first step in SLY-pore formation is the initial binding of the toxin monomer to the host cell, the amount of membrane-bound SLY might differ among the tested cell types, leading to a varying extent of cell damage. Thus, membrane binding capacity of SLY to the different cell types was determined using flow cytometry analysis (Figure 3A,B) after treatment of cells with 120 HU/mL rSLY for 2 h at 37 °C. NPTr cells showed the highest percentage of cells positive for SLY (75%) in comparison to HEp-2 cells (50%), however this difference was not significant. In contrast, in primary cells, significantly less (only 10%–15%) cells were positive for bound SLY (Figure 3A). Analysis of the mean fluorescence intensity revealed that the number of SLY-molecules bound per cell did not differ among the cell types (Figure 3B). However, it is not possible to conclude on the distribution of SLY-molecules and the presence of toxin monomers, oligomers, prepore-complexes, or fully expanded pores, respectively, by this analysis. Nevertheless, the low membrane binding ability of SLY to PTEC and PBEC might be the reason for the low susceptibility of both cell types. In HEp-2 and NPTr cells, the total amount of membrane-bound SLY *per se* does not seem to be crucial for the extent of SLY-induced cell damage. It may be assumed that on HEp-2 cells, more functional pores are formed, whereas in NPTr cells, the number of SLY-molecules is similar, however these may be distributed more homogenously and thus, complete pores are less frequent.

For SLY, as for other members of the CDC family, membrane binding and cytolytic activity is dependent on the presence of membrane cholesterol [61,62,63,64]. Furthermore, soluble cholesterol can inhibit cytolytic activity of SLY, which led to the assumption that cholesterol interferes with the biological activity of this toxin [15,33,39,46]. Therefore, we investigated the amount of cellular cholesterol present in the different cell types tested. For this, lipid extraction of cells was conducted and the proportion of cholesterol was analyzed using TLC and HPLC, respectively. In both cases, the amount of cholesterol was related to the total cellular protein content of the respective cells. TLC (Figure 3C) and HPLC (Figure 3D) revealed no significant difference in the cholesterol–protein ratios of HEp-2 and NPTr cells, respectively. However, PTEC and PBEC contained significantly lower amounts of cellular cholesterol as compared to the cell lines. This may explain the low membrane binding ability of SLY to those cells and thus, their low toxin susceptibility. Nevertheless, a higher amount of cellular cholesterol is not *per se* associated with higher membrane binding of SLY. It is more likely that a critical amount of cholesterol is necessary for efficient CDC membrane binding [63,64]. At a certain cholesterol threshold, a further increase does not lead to higher CDC binding [65]. Furthermore, in addition to the role of the (critical) amount of membrane cholesterol for binding of CDC, the composition of the lipid rafts [66,67], as well as the structure of cholesterol, is of importance [66,68,69]. Other studies showed that loss or depletion of membrane cholesterol reduces cytolytic pore formation by blocking the prepore-to-pore transition rather than by inhibiting the membrane binding of the CDC [13,70]. Moreover, although cholesterol may function as a receptor for most CDC [71,72,73], a specific cell-receptor for SLY is yet unknown. Notably, certain other CDC were reported to bind to human CD59, a GPI-anchored protein within the lipid rafts [9,10,11], as mentioned above. However, CD59-specific CDC also require cholesterol for the membrane insertion of the prepore-complex [12,13,14]. Taken together, currently the exact role of membrane cholesterol for membrane binding and cytolytic activity of CDC like SLY remains to be elucidated. Our results revealed that in NPTr and HEp-2 cells, the amount of cellular cholesterol is not the sole factor that is involved in the binding of SLY and the amount of membrane-associated SLY is not directly correlated with the extent of SLY-induced cell damage. In contrast, PTEC and PBEC showed a significant lower cellular cholesterol content, which correlated with a lower binding affinity of SLY and remarkably low damage induced by SLY.

### 2.3. SLY-Induced Cell Damage Can Be Repaired by Resealing in a Ca^2+^-Dependent Manner

**Figure 4:**
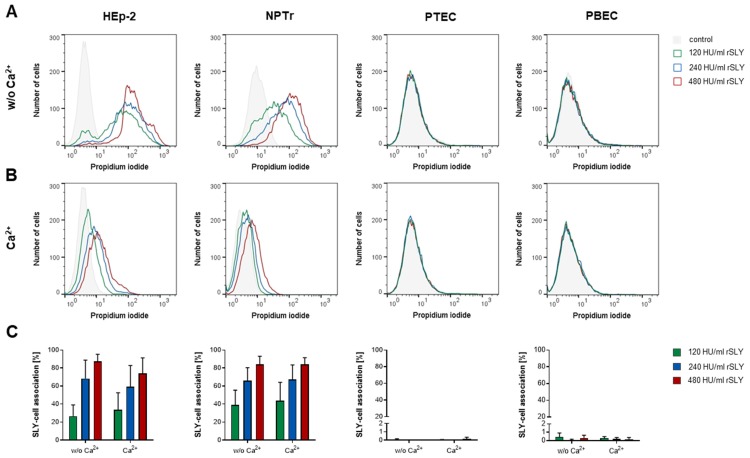
HEp-2 and NPTr cells, as well as PTEC and PBEC, were treated with 120–480 HU/mL rSLY for 5 min at 4 °C in the absence of Ca^2+^, followed by incubation for 5 min at 37 °C in the (**A**) absence or (**B**) presence of Ca^2+^. Cell damage was analyzed using flow cytometry analysis. A representative histogram for each cell type and treatment of at least three independent experiments is depicted, showing the number of cells positive for PI. Additionally, binding of SLY was analyzed and results are expressed as (**C**) percentage SLY-cell association. Mean ± SD of at least three independent experiments are shown.

Since membrane binding of SLY and the cholesterol content of cells were not the only factors responsible for the different susceptibilities of the tested epithelial cells, we considered another factor, the removal (resealing) of pores, as a possible mechanism to prevent or reduce cell damage. It is known that several cell types have the ability to reseal cytolysin-induced pores by different mechanisms in a calcium (Ca^2+^)-dependent manner [52,53,74]. Therefore, we speculated that the cells that were used in our experiments can recover from SLY-induced cell damage in a similar way. To test this hypothesis, HEp-2 and NPTr cells as well as undifferentiated PTEC and PBEC were treated with different concentrations of rSLY (120–480 HU/mL) for 5 min at 4 °C in the absence of Ca^2+^ to allow the toxin to bind to and build pores in the cell membrane. Afterwards, cells were incubated for 5 min at 37 °C either in the presence or in the absence of Ca^2+^. During this time, we expected the pores to be removed only when Ca^2+^ was available. Binding of SLY and the extent of cell damage, as measured by counting the number of cells positive for propidium iodide (PI), was analyzed using flow cytometry analysis. We found a dose-dependent cell damage in HEp-2 and NPTr cells that was reversible when Ca^2+^ was available (Figure 4A,B). However, resealing efficiency was dependent on the toxin concentration, i.e., cells treated with 480 HU/mL rSLY did not completely recover in contrast to cells treated with 120 HU/mL rSLY. Most strikingly, no differences between HEp-2 and NPTr cells were found. In undifferentiated primary cells, we could not detect any cells positive for PI whether Ca^2+^ was present or not. Determination of SLY-cell association revealed a dose-dependent binding of the toxin to HEp-2 and NPTr cells, independent of the presence of Ca^2+^ (Figure 4C). In PTEC and PBEC, almost no cells positive for SLY were detectable. This might explain the lack of damage observed in those cells, consistent with the results for PTEC and PBEC described above. Since the number of cells positive for SLY was not reduced by the resealing process, we assume that resealing can remove pores, thereby blocking the influx of PI. However, elimination of all SLY monomers, oligomers, or prepore-complexes is, most likely, not possible during this short incubation time. It should be emphasized that under the conditions of this experiment (absence of Ca^2+^, short incubation time, 4 °C), in contrast to the binding study described above, both HEp-2 and NPTr cells showed the same binding affinity of SLY, as well as the same susceptibility towards the toxin. However, it has to be considered that the absence of extracellular Ca^2+^ facilitates the binding and/or assembly of the toxin monomers [75] and, additionally, the repair machinery cannot be initiated when Ca^2+^ is not available [76,77]. Taken together, these data show that NPTr and HEp-2 cells are able to reseal SLY-induced pores rapidly in a Ca^2+^-dependent manner.

### 2.4. NPTr Cells Reseal SLY-Induced Cell Damage More Efficiently Than HEp-2 Cells

**Figure 5:**
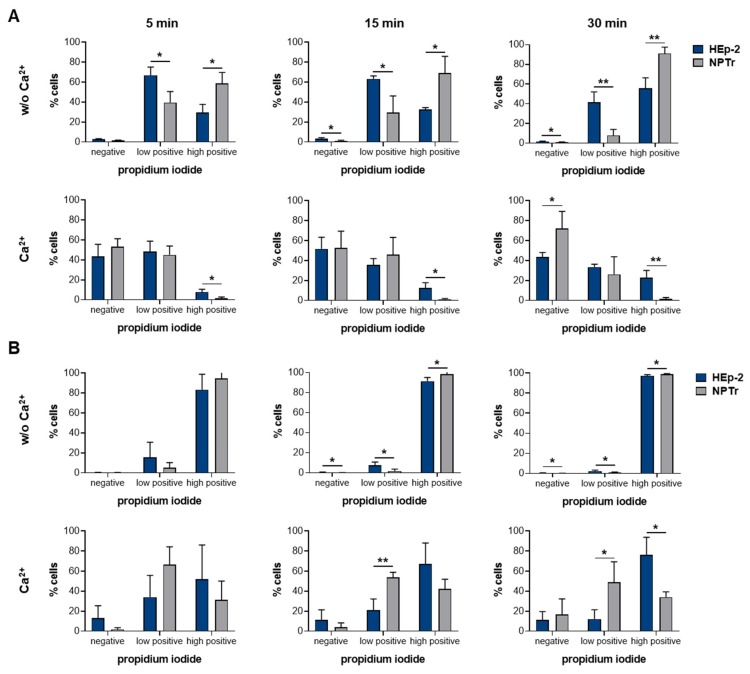
HEp-2 and NPTr cells were treated with (**A**) 120 HU/mL and (**B**) 480 HU/mL rSLY for 30 min at 4 °C in the absence of Ca^2+^, followed by incubation for 5, 15, and 30 min at 37 °C in the absence or presence of Ca^2+^. Cell damage was analyzed using flow cytometry analysis. Results are expressed as the percentage of cells negative, low positive, and high positive for PI and mean ± SD of at least three independent experiments are shown. Significant differences between the cell types are indicated as * *p* < 0.05 and ** *p* < 0.01, *t*-test.

Membrane binding of SLY and the capacity to remove pores caused by SLY does not fully explain the differences between the susceptibility of HEp-2 and NPTr cells towards the toxin. Thus, we hypothesized that NPTr cells can restore the membrane integrity in a more efficient and/or sustained way than HEp-2 cells. To investigate this theory, we modified the experiment described above. Treatment of cells with 120 HU/mL rSLY (Figure 5A) and 480 HU/mL rSLY (Figure 5B) was extended to 30 min at 4 °C in the absence of Ca^2+^, followed by incubation at 37 °C in the presence or absence of Ca^2+^ up to 30 min. Then, we determined the cells which were negative, low positive, and high positive for PI using flow cytometry analysis (gating Figure S2). When comparing HEp-2 and NPTr cells, we found significant differences in the resealing capacity. In particular, after treatment with 480 HU/mL rSLY, more NPTr cells than HEp-2 cells were able to shift from the PI high population to the PI low or PI negative population (Figure 5B). The same was found for the lower concentrations of rSLY (Figure 5A). Nevertheless, longer incubation time at 37 °C in the presence of Ca^2+^ (15 and 30 min) did not substantially improve the resealing efficiency. Interestingly, NPTr cells showed a higher sensitivity towards the toxin in the absence of Ca^2+^ compared to HEp-2 cells, as more NPTr cells were highly positive for PI when treated with 120 HU/mL rSLY. However, NPTr cells seem to have a higher capacity to recover from SLY-induced cell damage during long term incubation with SLY than HEp-2 cells as a higher number of NPTr cells moved to the PI low or even PI negative population when Ca^2+^ was available. In good agreement with the results shown before, we found that resealing capacity is critically dependent on the toxin concentration and the related cell damage. Notably, when a certain level of damage is induced, cells are not able to recover completely, which could be associated with a critical level of Ca^2+^ influx [76]. Taken together, the efficiency of cells to remove SLY-induced pores seems to be the critical factor to prevent cell lysis, which explains why HEp-2 cells are more susceptible to SLY than NPTr cells.

Thus, future studies should address the resealing mechanisms and efficiencies of different host cells since these might be important for protection against SLY-induced cell damage. In case of SLO, the archetype of a CDC produced by *S. pyogenes*, pores are repaired by Ca^2+^-dependent and annexin-mediated fusion of the plasma membrane and shed as microvesicles into the extracellular space (ectocytosis) [76,77,78]. The same mechanism of Ca^2+^-dependent microvesicle shedding was reported for the CDC listeriolysin O (LLO), perfringolysin O (PFO), intermedilysin (ILY), and PLY [79,80,81]. Another possibility to prevent cell lysis is the Ca^2+^-dependent endocytic removal and internal degradation of the plasmalemmal lesion, which has also been described for SLO [52], as well as for other pore-forming toxins [82,83]. Furthermore, both mechanisms can occur simultaneously, whereas microvesicle shedding facilitates the initial elimination of the toxin pores and the lysosome-mediated endocytic removal deals with the secondary mechanical injury [84]. Which type of repair mechanism eventually occurs depends on the cell type, the extent of the membrane lesion, and the causing agent, as well as the Ca^2+^-concentration [56,74,84].

## 3. Conclusions

This study showed that porcine respiratory epithelial cells, including the porcine respiratory cell line NPTr and undifferentiated primary porcine respiratory epithelial cells, are less sensitive towards the activity of SLY when compared to the human epithelial cell line HEp-2, which is frequently used in studies on the biological role of SLY and other CDC. In the case of the two respiratory cell lines, higher cytotoxic effects were not associated with higher membrane binding of SLY and the amount of cellular cholesterol was not directly correlated with membrane binding of SLY. However, concerning the resealing capacity, we found that NPTr cells can reseal SLY-induced cell damage more efficiently than HEp-2 cells, in particular when the cell damage was more pronounced due to higher toxin concentrations or longer treatment time. However, more studies in the future are needed to identify the precise role of membrane composition, receptor expression, and resealing mechanism(s) in the susceptibility and/or protection of host cells to SLY and CDC in general. Finally, the substantial differences in susceptibility to SLY-induced damage of the cell lines as compared to primary respiratory cells underlines that in vitro studies on biological functions of virulence-associated factors, such as SLY, should be performed with models which more closely represent the in vivo situation than permanent cell line models. Hence, we suggest that future studies focus mainly on models consisting of differentiated respiratory epithelial cells, such as air-liquid interface cultures and precision-cut lung slices. These models are suitable to fill the gap between permanent cell lines and animal models. Furthermore, we want to emphasize the importance of choosing the proper host species as previous studies showed that SLY is a critical virulence factor in the mouse model [16,85,86], but not in pigs [31,86].

## 4. Materials and Methods

### 4.1. Bacterial Strains and Recombinant Suilysin Protein

The virulent SLY-positive *S. suis* serotype 2 wild-type (WT) strain was kindly provided by H. Smith (Lelystad, NL) [87]. Its isogenic SLY-deficient mutant (∆sly) was constructed by the insertion of an erythromycin cassette in the *sly* gene [88]. Both strains were grown on Columbia agar supplemented with 7% (*v/v*) sheep blood (Oxoid™, Thermo Fisher Scientific, Waltham, MA, USA) overnight at 37 °C under aerobic conditions. For infection experiments, cryo-conserved bacterial stocks were prepared from liquid cultures in Todd-Hewitt Broth (THB; Bacto™, Becton Dickinson, Heidelberg, Germany) at the late-exponential growth phase (OD_600_ 1.1) as previously described [24].

The recombinant SLY (rSLY) protein was expressed in *Escherichia coli* BL21 (DE3) and purified as described before [88,89]. The purified protein was controlled by immunoblot analysis and the concentration was determined by *DC*™ Protein Assay (Bio-Rad Laboratories, Munich, Germany). The protein was stored at −80 °C.

### 4.2. Cell Culture

We used the human laryngeal epithelial cell line HEp-2 (ATCC^®^, CCL-23™) and newborn pig tracheal epithelial cells (NPTr); the latter were kindly provided by F. Meurens (Nantes, France) [50]. HEp-2 cells were cultured in Dulbecco’s Modified Eagle Medium (DMEM; Thermo Fisher Scientific, Waltham, MA, USA) supplemented with 10% (*v/v*) fetal calf serum (FCS; Biochrom, Berlin, Germany) and 1% (*v/v*) L-glutamine (Thermo Fisher Scientific, Waltham, MA, USA) at 37 °C and 8% CO_2_ in a humidified atmosphere. NPTr cells were cultured in DMEM supplemented with 5% (*v/v*) FCS.

Primary porcine tracheal (PTEC) and bronchial (PBEC) epithelial cells were isolated from swine lungs obtained from a local slaughterhouse (Leine-Fleisch GmbH, Laatzen, Germany), as described before [44]. Epithelial cells were cultured in collagen I (Sigma-Aldrich, Taufkirchen, Germany)-coated T75 cell culture flasks in Bronchial Epithelial Cell Basal Medium (BEBM™; Lonza, Basel, Suisse) supplemented with several growth factors (Bronchial Epithelial Cell Growth Medium, BEGM) [90] and antibiotics (100 U/mL penicillin, 0.1 mg/mL streptomycin, 2.5 µg/mL amphotericin B, 50 µg/mL gentamycin) at 37 °C and 5% CO_2_ in a humidified atmosphere.

### 4.3. Cytotoxicity Assay

The cytotoxic activities of the two *S. suis* strains (WT and Δsly) and of the rSLY towards the different cells used in this study were determined by measuring the release of lactate dehydrogenase (LDH) using CytoTox 96^®^ Non-Radioactive Cytotoxicity Assay (Promega, Mannheim, Germany). For this purpose, HEp-2 and NPTr cells, as well as undifferentiated PTEC and PBEC, were seeded on 24-well tissue culture plates (Greiner Bio-One, Frickenhausen, Germany) and confluent cells were incubated with *S. suis* WT and Δsly, respectively, at MOI 100:1 for 2 h and 4 h at 37 °C. Accordingly, for the treatment with rSLY cells, they were seeded on 96-well tissue culture plates (Sarstedt, Nümbrecht, Germany) and confluent cells were incubated with 300–19,200 HU/mL rSLY for 2 h and 4 h, respectively, at 37 °C. After incubation, supernatants were removed and LDH release assay was performed. All experiments were performed in duplicates and repeated at least three times. Results were expressed as percentage LDH release compared to Triton^®^ X 100 (Carl Roth, Karlsruhe, Germany) lysed non-infected/-treated cells.

### 4.4. Immunoblot Analysis

Supernatants of cells infected with *S. suis* WT and Δsly were separated electrophoretically using a 5% (*v/v*) stacking and a 10% (*v/v*) running SDS-polyacrylamide gel and were transferred to a PVDF-membrane (Merck Millipore, Darmstadt, Germany). The membranes were blocked for one hour at RT with 5% (*v/v*) milk powder in Tris-buffered saline (TBS) with 1% (*v/v*) Tween^®^ 20 (Carl Roth, Karlsruhe, Germany) and incubated with polyclonal antiserum raised against rSLY [88] (diluted 1:1000 in 1% (*v/v*) milk powder in TBS with 1% Tween^®^ 20) overnight at 4 °C to detect rSLY. Development of the membranes was performed with AP-conjugated goat anti-rabbit IgG (Jackson Immuno Research, West Grove, PA, USA) (diluted 1:10,000 in 1% milk powder in TBS with 1% Tween^®^ 20, incubated for 1 h at RT), AP juice (PJK, Kleinblittersdorf, Germany), and chemiluminescence detection with ChemoCam Imager 3.2 (Intas, Göttingen, Germany).

### 4.5. FACS Analysis of SLY-Cell Association

To determine membrane binding of SLY to HEp-2 and NPTr cells, as well as undifferentiated PTEC and PBEC, 4 × 10^5^ trypsinized cells were incubated with 120 HU/mL rSLY in 1 mL cell culture medium for 2 h at 37 °C. Cells were washed with PBS (Sigma-Aldrich, Taufkirchen, Germany) with 2% (*v/v*) FCS (Biochrom, Berlin, Germany) and stained using polyclonal antiserum raised against rSLY [88] (diluted 1:1000 in PBS with 2% FCS, incubated for 1 h at RT) and Alexa Fluor^®^ 488 goat-anti-rabbit IgG antibody (Thermo Fisher Scientific, Waltham, MA, USA) diluted 1:1000 in PBS with 2% FCS, incubated for 30 min at RT). Additionally, cells were stained with the DNA-intercalating dye propidium iodide (PI, 2.5 µg/mL; Sigma-Aldrich, Taufkirchen, Germany) for 5 min at RT to discriminate viable and non-viable cells. SLY-cell association was measured using Guava^®^ EasyCyte8 (Merck Millipore, Darmstadt, Germany). The cell population was identified using forward- and side-scatter light, and green- and red-fluorescent cells were detected. In all the experiments, at least 5000 events were counted and analyzed with FlowJo software version 10.5.2 (Tree Star Inc., Ashland, OR, USA). The experiment was repeated at least three times. Results are expressed as percentage SLY-cell association and mean fluorescence intensity.

### 4.6. Lipid Extraction and Quantitative Measurement of the Cellular Cholesterol and Protein Content

For lipid extraction, 2 × 10^6^ cells of HEp-2 and NPTr cells, as well as undifferentiated PTEC and PBEC, were resuspended in methanol (M; Carl Roth, Karlsruhe, Germany) and sonicated at 4 °C for 4 × 40 s (Sonifier 450, Branson, Danbury, Connecticut). Afterwards, chloroform (C; Carl Roth, Karlsruhe, Germany) was added (final solvent mixture C/M 2:1) and the mixture was stirred overnight. Supernatant was collected by centrifugation and evaporated at RT [91]. For determining cellular cholesterol content by thin-layer chromatography (TLC), lipids were dissolved in 100 µL C/M 2:1. Two µl of the lipid solution were loaded on a TLC plate (DC-Fertigfolien ALUGRAM Xtra SIL G/UV; Macherey-Nagel, Düren, Germany) and cholesterol standard solutions were included. The chromatography was performed in a TLC chamber saturated with C/M 94:6. Afterwards, the TLC plate was stained with Hanessian’s stain [92], baked for 1 min at 150 °C, and scanned at 300 dpi. Finally, the signal was quantified using the software LabImage 1D (Intas, Göttingen, Germany). Additionally, lipids extracted from the different cell types were analyzed with high performance liquid chromatography (HPLC) as described previously [93]. An external standard was used to quantify the amount of cholesterol per million cells. The cellular cholesterol content was related to the total cellular protein. For this, cell extraction buffer (Thermo Fisher Scientific, Waltham, MA, USA) with 0.5 mM AEBSF (Merck Millipore, Darmstadt, Germany) and protease inhibitor (diluted 1:10; Sigma-Aldrich, Taufkirchen, Germany) was added to the remaining cell debris from the lipid extraction. Cells were lysed in a bead beater for 5 × 40 s at full speed. The supernatant was collected by centrifugation and *DC*™ Protein Assay (Bio-Rad Laboratories, Munich, Germany) was carried out to determine the amount of protein. This experiment was repeated at least three times. Results are expressed as a cholesterol–protein ratio.

### 4.7. FACS Analysis of Membrane Resealing Capacity

To investigate the membrane resealing capacity of HEp-2 and NPTr cells, as well as undifferentiated PTEC and PBEC, we performed an assay modified from Idone et al. [52]. Briefly, 4 × 10^5^ trypsinized cells were treated with 120–480 HU/mL rSLY for 5 min or 30 min at 4 °C in calcium (Ca^2+^)-free DMEM (Thermo Fisher Scientific, Waltham, MA, USA) supplemented with 10% (*v/v*) FCS (Biochrom, Berlin, Germany), 1% (*v/v*) L-glutamine (Thermo Fisher Scientific, Waltham, MA, USA), and 5 mM EGTA (Sigma-Aldrich, Taufkirchen, Germany) (referred to as “DMEM w/o Ca^2+^”). Subsequently, the medium was changed to either DMEM supplemented with 10% (*v/v*) FCS and 1% (*v/v*) L-glutamine (referred to as “DMEM + Ca^2+^”) or DMEM w/o Ca^2+^ and cells were incubated for 5, 15, or 30 min at 37 °C. Afterwards, cells were stained with 5 µg/mL PI (Sigma-Aldrich, Taufkirchen, Germany), polyclonal antiserum raised against rSLY [88] (diluted 1:1000 in PBS with 2% FCS, for 1 h at RT), and Alexa Fluor^®^ 488 goat-anti-rabbit IgG antibody (Thermo Fisher Scientific, Waltham, MA, USA; diluted 1:1000 in PBS with 2% FCS, for 30 min at RT). Cell damage and SLY-cell association were measured using Guava^®^ EasyCyte8 (Merck Millipore, Darmstadt, Germany). The cell populations were identified using forward- and side-scatter light, and green- and red-fluorescent cells were detected. In all experiments, at least 5000 events were counted and analyzed with FlowJo software version 10.5.2 (Tree Star Inc., Ashland, OR, USA). The experiment was repeated at least three times. Results are presented in histograms showing cells positive for PI (Figure 4A,B) or are expressed as cells negative, low positive, and high positive for PI (Figure 5, gating Figure S2), respectively, or as percentage SLY-cell association (Figure 4C).

### 4.8. Statistical Analysis

All experiments were repeated at least three times and data in the figures are shown as the means ± standard deviation (means ± SD). All statistical analyses were carried out using GraphPad Prism version 8.0.1 for Windows (GraphPad Software, San Diego, CA, USA). Statistical significance was determined by either one-way or two-way ANOVA followed by Tukey post-hoc test or by *t*-test; *p* < 0.05 was considered to be statistically significant.

## Supplementary Materials

The following are available online at https://www.mdpi.com/2076-0817/9/1/33/s1, Figure S1: Growth of *S. suis* in the presence of different epithelial cells; Figure S2: Time-dependent resealing of SLY-induced cell damage. One exemplary histogram for each cell type is depicted, showing the gating of cells negative, low positive, and high positive for propidium iodide, respectively.
