# Supplementary material for: Membrane Binding, Cellular Cholesterol Content and Resealing Capacity Contribute to Epithelial Cell Damage Induced by Suilysin of Streptococcus suis
Source: Pathogens. 2019 Dec 30;9(1):33. doi: 10.3390/pathogens9010033 (PMC7168673; doi:10.3390/pathogens9010033)
Supplement: Supplementary file 1 [file pathogens-09-00033-s001.pdf]

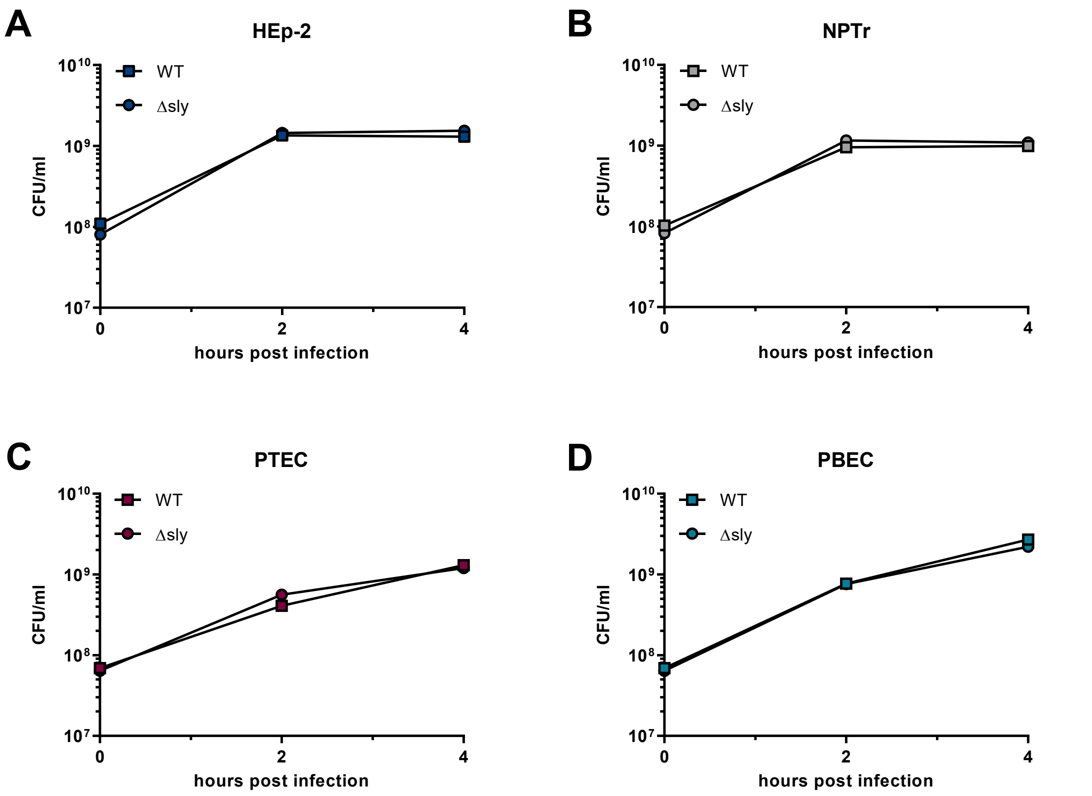


**Figure S1.** (A) HEp-2 and (B) NPTr cells, as well as (C) PTEC and (D) PBEC, were incubated with *S. suis* wild-type (WT) strain 10 and its SLY-deficient mutant (Δ*sly*) at MOI 100:1 for up to 4 h at 37°C. Growth kinetics of *S. suis* were determined by counting of colony forming units (CFU)/ml after serial dilution of the supernatant of infected cells and plating on blood agar plates. Results of at least one representative experiment are shown.

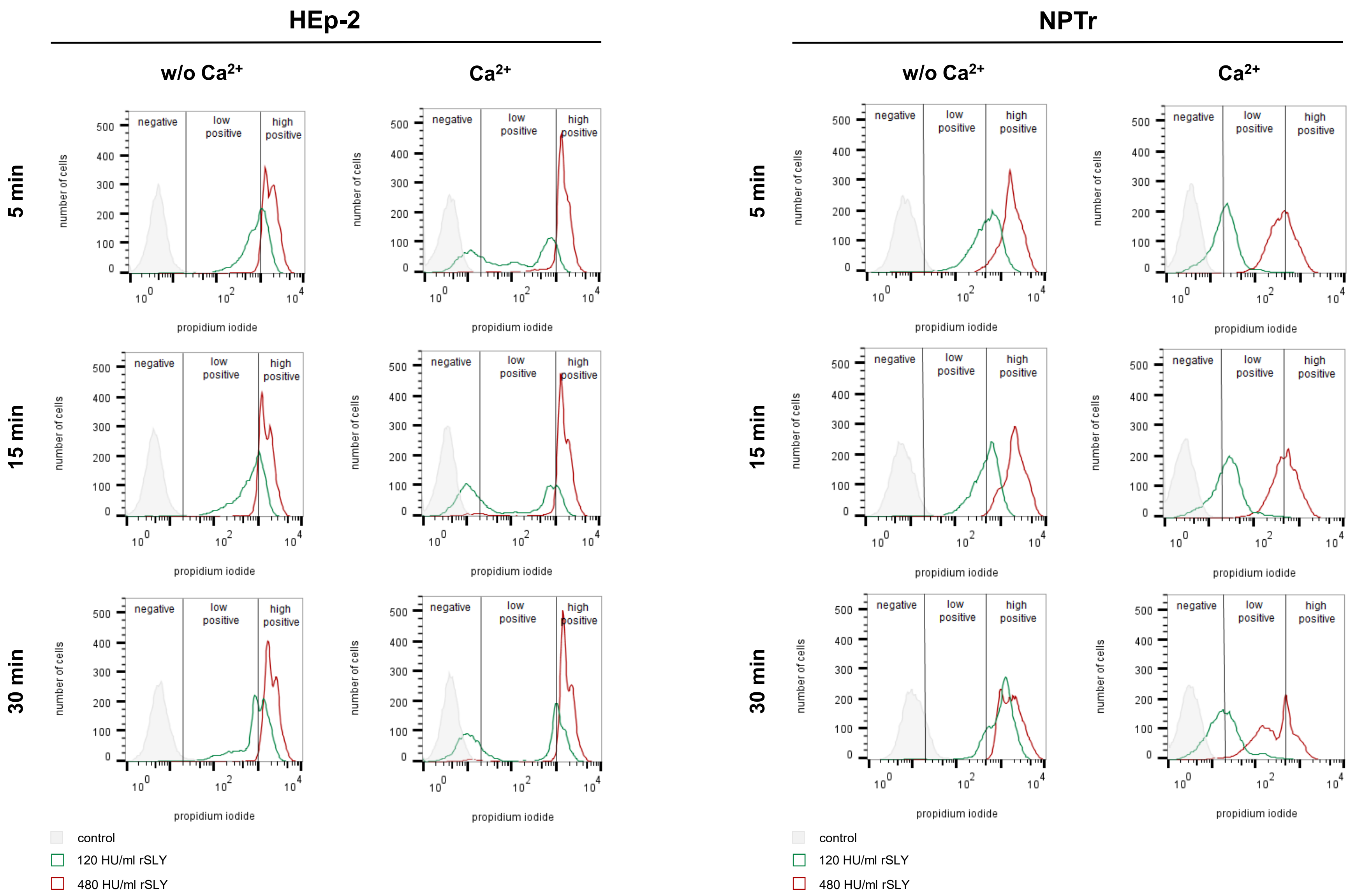


**Figure S2.** HEp-2 and NPTr cells were treated with 120 and 480 HU/ml rSLY for 30 min at 4°C in the absence of $Ca^{2+}$, followed by incubation for 5, 15, and 30 min at 37°C in the absence or presence of $Ca^{2+}$. Cell damage was analyzed using flow cytometry. One exemplary histogram for each cell type of at least three independent experiments is depicted, showing the gating of cells negative, low positive and high positive for PI, respectively.
